# Clinicians' Perspectives on Racism and Black Women's Maternal Health

**DOI:** 10.1089/whr.2021.0148

**Published:** 2022-05-04

**Authors:** Brittany D. Chambers, Brianne Taylor, Tamara Nelson, Jessica Harrison, Arielle Bell, Allison O'Leary, Helen A. Arega, Sepehr Hashemi, Safyer McKenzie-Sampson, Karen A. Scott, Tina Raine-Bennett, Andrea V. Jackson, Miriam Kuppermann, Monica R. McLemore

**Affiliations:** ^1^ Department of Human Ecology, College of Agricultural and Environmental Sciences, University of California, Davis, Davis, California, USA.; ^2^ California Preterm Birth Initiative, School of Medicine, University of California, San Francisco, San Francisco, California, USA.; ^3^ Department of Family Health Care Nursing, School of Nursing, University of California, San Francisco, San Francisco, California, USA.; ^4^ Department of Social and Behavioral Sciences, School of Nursing, University of California, San Francisco, San Francisco, California, USA.; ^5^ Department of Obstetrics, Gynecology and Reproductive Sciences, School of Medicine, University of California, San Francisco, San Francisco, California, USA.; ^6^ Department of Epidemiology and Biostatistics, School of Medicine, University of California, San Francisco, San Francisco, California, USA.; ^7^ Department of Humanities and Social Sciences, University of California, San Francisco, San Francisco, California, USA.; ^8^ Medicines360, San Francisco, California, USA.

**Keywords:** African Americans, maternal health, racism, women's health

## Abstract

**Objective:**

The objective of this study was to explore clinician perceptions of how racism affects Black women's pregnancy experiences, perinatal care, and birth outcomes.

**Materials and Methods:**

We conducted 25 semi-structured interviews with perinatal care clinicians practicing in the San Francisco Bay Area (January to March 2019) who serve racially diverse women. Participants were primarily recruited through “Dear Perinatal Care Provider” email correspondences sent through department listservs. Culturally concordant, qualitatively trained research assistants conducted all interviews in person. The interviews ranged from 30 to 60 minutes and were audio-recorded and professionally transcribed verbatim. We used the constant comparative method consistent with grounded theory to analyze data.

**Results:**

Most participants were obstetrician/gynecologists (*n* = 11, 44%) or certified nurse midwives (*n* = 8, 32%), had worked in their current role for 1 to 5 years (*n* = 10, 40%), and identified as white (*n* = 16, 64%). Three themes emerged from the interviews: provision of inequitable care (*e.g.*, *I had a woman who had a massive complication during her labor course and felt like she wasn't being treated seriously*); surveillance of Black women and families (*e.g.*, *A urine tox screen on the Black baby even though it was not indicated, and they didn't do it on the white baby when, in fact, it was indicated*); and structural care issues (*e.g.*, *the history of medical racial experimentation*).

**Conclusion:**

Clinicians' views about how racism is currently operating and negatively impacting Black women's care experiences, health outcomes, and well-being in medical institutions will be used to develop a racial equity training for perinatal care clinicians in collaboration with Black women and clinicians.

## Introduction

Inequities in adverse maternal and child health outcomes persist in the United States. Black women are three to four times more likely to experience complications during pregnancy and childbirth and die from these complications compared with white women.^1–3^ Additionally, infants born to Black women are two times more likely to be born premature (<37 weeks of gestation) compared with infants born to white women.^4^ Care provided during pregnancy and childbirth can potentially reduce complications by aiding in the early detection and successful management of pre-existing or newly diagnosed comorbid medical conditions.^5–9^ Despite the benefits of care during pregnancy and childbirth, racism serves as a barrier to Black women accessing and receiving quality care.^5–8,10^

Racism is a multifaceted system developed as a tool to classify groups of people based on physical attributes and characteristics such as skin color, serving to oppress Black people.^11,12^ To sustain white supremacy in the United States, laws (*e.g.*, Jim Crow Laws) and processes (*e.g.*, Redlining) were established to allocate resources in ways that increased wealth and opportunity to whites.^11,12^ Promulgating negative stereotypes and scripts about Black people has added to these barriers to attaining and sustaining their economic wealth, bodily autonomy, and liberal freedoms.^11^

Stereotypes are perceived notions of a person based on social and physical attributes, whereas scripts are ways people are taught or believe they should interact with people based on their physical and social attributes.^13,14^ In the context of reproductive health, racism was used as a tool to establish therapies and surgical techniques in the field of gynecology.^15^ In the 19th century, J. Marion Sims performed experimental surgery on enslaved Black women without their consent to develop a cure for vesicovaginal fistula.^15^ These experiments facilitated the generations of two key health care scripts about Black women in the context of reproductive health care: (1) it is acceptable to perform procedures on Black women without their consent; and (2) Black women have a high tolerance for pain.^16,17^

Institutionalized racism in health care settings contributes to Black women receiving lower quality of care in comparison to white women, resulting in inequities in adverse maternal and child health outcome.^18–24^ There is an association between racial discrimination and Black women's dissatisfaction with care, mistrust in providers, non-adherence to medical regimes, and ineffective patient-provider communication.^5–7,25,26^ Studies have also shown that poor quality of care is associated with Black women being offered fewer or mistimed treatments and interventions.^27–29^ Research supports that any approach to improving Black women's quality of care must address clinicians' potential racial biases and racism towards Black people.^30–34^

Clinicians' biases and racism have been shown to be associated with poor health outcomes among Black compared with white patients.^7,26,30–33,35^ van Ryn et al.^31^ developed a conceptual framework of racism and how it impacts clinicians' cognition, and clinical decision-making, conveying how structural and institutional racism contributes to clinicians' implicit and explicit racial biases that then inform clinicians' communication, screening, diagnostic, and treatment behaviors. This foundational work has led to a call for action for health care and grant-making institutions to address structural factors in an effort to improve health outcomes for Black, Indigenous, and other people of color, proposing a move from cultural to structural competency.^36,37^

Curriculums and praxis tailored toward medical students and the field of obstetrics and gynecology have used structural competency^38^ and justice frameworks^19^ to understand and address racism in prenatal and delivery care settings, with a few curriculums recently developed to focus on clinicians since California's new legislation (AB 241 and SB 464) requires continuing education in implicit bias.^37,39,40^ Although there is a plethora of research documenting Black women's experiences of racism and discrimination while navigating perinatal care, much less has been reported regarding the relationship between racism and clinical care through the lens of clinicians' caring for Black women during pregnancy and childbirth.

In preparation for the development of a new curriculum, the objective of this study was to explore clinicians' perceptions of how racism affects Black women's perinatal experiences and outcomes. Research suggests that effective components of racial and cultural equity trainings include presenting data that are relevant to clinicians, providing vignettes of real-life experiences, sharing tools that can be used in clinic, and implementing quality improvement metrics.^41–43^ Our study participants' stories will be used to develop vignettes of real-life racist events that have occurred in perinatal care settings to guide clinicians through activities to help them critically think about anti-racist approaches to providing care.

## Materials and Methods

We conducted a qualitative research study with a convenience sample of 25 perinatal care clinicians. Clinicians were eligible to participate in the study if they were currently providing perinatal care at one of two California-based hospital facilities in the San Francisco Bay Area serving racially diverse women. We recruited clinicians to participate in the study through “Dear Perinatal Care Clinician” email correspondence sent through department listservs and *via* presentations at department meetings. We used a semi-structured interview guide to conduct one-on-one interviews with participants, who provided informed consent and received a $50 American Express gift card for their participation.

The interview guide focused on clinicians' perspectives about the leading factors associated with the persistent racial equity gap between Black and white women in maternal and child health outcomes, the role racism may play in these inequities, and the content and activities these clinicians would like to see in a racial equity training to improve Black women's perinatal care experiences and outcomes. Interviews were conducted in-person between January and March 2019 by culturally concordant research assistants trained in qualitative research methods. Interviews ranged between 30 and 60 minutes and were audio-recorded and professionally transcribed.

The study team met on a weekly basis during the data collection process to discuss interview findings and emerging themes. Each research assistant wrote field notes within 24 hours of completing each interview to enable them to reflect on their interview experience and summarize key themes and ideas that emerged. Data collection was stopped once saturation across key themes and ideas was reached. Upon completion of the interviews, the study team reviewed transcripts and collectively developed a codebook for data analysis. The study team used modified grounded theory techniques, specifically an iterative process of coding and analysis and the constant comparative method, to identify codes and sort them into larger themes.^44,45^

Two research assistants coded each transcript, with an inter-rater reliability of 93%. All discrepancies were discussed, and final codes were determined collectively as a team. Quotes throughout this article are labeled based on clinicians' facility (*e.g.*, 01), interview order for each facility (*e.g.*, 010), role (*e.g.*, obstetricians/gynecologists [OB/GYN]), and race and/or ethnicity and gender identity (*e.g.*, Black identified person). For example, a label such as “01-010, OB/GYN, Black identified person” will follow each quote. All data were coded and analyzed using Dedoose Version 7.0.23 (Los Angeles, CA, USA). This study was approved by the institutional review board at the University of California, San Francisco (#18-26494).

## Results

Characteristics of our study participants are provided in Table 1. The 25 perinatal care clinicians we interviewed included OB/GYN (*n* = 11, 44%), certified nurse midwives (CNM) (*n* = 8, 32%), family medicine practitioners (*n* = 2, 8%), and fellows/residents (*n* = 4, 16%). On average, these clinicians had worked in their current role for 8.9 years. The majority of the participants identified as white (*n* = 16, 64%) and women (*n* = 23, 92%).

**Table 1.** Clinicians' Characteristics

Characteristic	*n* (%)
Role	
Certified nurse midwife	8 (32)
Obstetrician/gynecologist	11 (44)
Family medicine practitioner	2 (8)
Fellow/residents	4 (16)
Time in role (years)	
<1	3 (12)
1–5	10 (40)
6	4 (16)
>10	8 (32)
Race/ethnicity	
African American/Black	4 (16)
Other^a^	5 (20)
White	16 (64)
Gender identity	
Woman	23 (92)
Man	2 (8)

^a^ Other race = Biracial, Middle Eastern, South Asian, and Latinx.

Participants shared three distinct ways in which racism impacts health care systems and care provided to Black women: (1) provision of inequitable care; (2) surveillance of Black women and families; and (3) structural care issues.

### Theme 1: provision of inequitable care

Provision of inequitable care was defined as racism influencing clinicians' ability to acknowledge and engage Black women as agents of their own bodies and the care provided by hospitals. Participants described that racism influences clinicians' racist stereotypes and beliefs about Black women, which results in inadequate care. An OB/GYN at Facility 1, for example, shared that clinicians' racist ideologies and beliefs about Black women directly influence not only the quality but also the frequency of care offered to Black women during their pregnancy and birthing experiences.

I think there are times when maybe Black women are assumed to be less compliant because of their race … or when we've had patients who have come in to triage frequently during their pregnancies … I think when providers are sort of stressed by that sort of recurrent presentation … if a patient is a minority, they may be more likely to sort of trigger people's [health care clinicians] bad behavior. (01-002, OB/GYN, white identified woman)

A CNM at Facility 2 shared how racist stereotypes and beliefs about Black women influence the type of care and treatment options offered to Black women in the postpartum period.

In the realm of family planning, or in the realm of lactation support, or in the realm of … pain management and labor, I think there are assumptions based on race that people make, like someone might assume … she's probably not going to breastfeed anyway so I'm maybe not going to go as far as I would go in my own commitment or my own perseverance in really being supportive. (02-005, CNM, white identified woman)

These quotes highlight perceptions that Black women inappropriately utilize health care—either too frequently or not enough—and assumptions about Black women's preferences about pregnancy, childbirth, and parenting, illustrating how racism is an oppressive system influencing clinicians' health care scripts about how to interact with and care for Black women.

Racism impacts clinicians' ability to listen to Black women's experiences and treat them as equal partners in decision-making about their own care and treatment options. A CNM at Facility 1 shared a personal account of her patient not being listened to while birthing at the hospital, noting that while her patient was in a life-threatening situation her expressed level of pain was disregarded.

I had a woman who … was actually my prenatal patient … she had a massive complication during her labor course and was in a lot of pain, felt like she wasn't being treated seriously. She had a life-threatening situation, and I felt like she was not being listened to. She was not. She was not being listened to … and she was very clear and very vocal while she was in this state. She kept saying, “This is why Black and Brown women die, because you're not listening to me.” (01-010, CNM, white identified woman)

Clinicians acknowledged that racism causes and impacts the provision of inequitable care provided to Black women, highlighting Black women are often dismissed and not included as active participants in care decisions and treatment.

### Theme 2: surveillance of Black women and families

Surveillance of Black women and families was defined as racism influencing clinicians' perceptions of Black women, children, and families, resulting in punitive care and treatment. The most common penalties shared among clinicians from both facilities were racial inequities in unwarranted urine toxicology (UTOX) screening or child protective services (CPS) involvement. For example, a CNM from Facility 2 shared that Black women are more often penalized for not showing up for prenatal care appointments than are white women:

I think that a white woman that misses five prenatal appointments probably isn't going to have a UTOX. The person of color is going to have a UTOX, like where have you been, what have you been doing? (02-002, CNM, white identified woman)

An OB/GYN from Facility 2 expressed similar concerns around racist treatment involving a UTOX screening that a Black infant received when a white infant in the same setting would not have had one.

When I was in labor and delivery attending a few times ago … There were two babies. One was white, one was Black, and they ended up doing a urine tox screen on the Black baby even though … according to the protocol, it was not indicated, and they didn't do it on the white baby when in fact, according to the protocol, it was indicated. (02-003, OB/GYN, white identified woman)

These stories underscore how clinicians' unethical UTOX screenings that do not align with protocols and procedures at their facilities negatively impact Black women's care.

A fellow/resident from Facility 2 expressed that excessive punitive and authoritative actions expand beyond Black women and include the policing of their support persons, particularly in birthing settings.

I've seen Black women who have had multiple family members who have tried to come visit them or they've had other support people come visit them or even pets or other people that are part of their community and more often those patients, their families and their people are seen as disruptive or they've been asked to sign contracts about the number of people who can be allowed in the room, which, to me, has no medical justification. (02-009, Fellow/Resident, Black identified woman)

These stories stress how clinicians perpetuate racial stereotypes of Black women, children, and families, which can cause harm and disrupt the structure of the Black family.

### Theme 3: structural care issues

Structural care issues were defined as the historical and contemporary impact of health care-based racism on Black women's reproductive health care experiences and outcomes. Participants expressed a heightened awareness of structural racism and how it impacts both Black women's health outcomes, medical education, and clinicians' interactions with patients. They shared that health care institutions in general, and the field of OB/GYN in particular, were established using racist procedures and protocols to advance the reproductive health of white women at the expense of Black women. An OB/GYN from Facility 2 shared their experiences in learning about and acknowledging institutional racism and the role it plays in the care clinicians provide to Black women.

We're learning, now, we've always known but it's being exposed more about the racism inherent in medicine from the history of medicine in the US, like Dr. Sims and gynecologists that were experimenting on Black women, as well as the Henrietta Lacks story … So trying to unpack how that translates to care that people receive or don't receive right now. (02-008, OB/GYN, white identified woman)

Clinicians acknowledged that the field of OB/GYN was built on racist ideologies, beliefs, language, and behaviors and that there is a need to dismantle the system to improve Black women's reproductive health experiences and outcomes. An OB/GYN from Facility 1 expressed struggling with training students, residents, and fellows on racial equity without changing how the current system values and serves Black women.

Despite the efforts around training trainees or providers in certain ways are we really challenging the underpinnings of a structure that is so oppressive and racist … I've just been thinking a lot about this in terms of we've now recruited this very diverse group of trainees, but we still educate them in the ways that we always have, which is in some ways perpetuating sort of white supremacy and hierarchy within our models. (01-006, OB/GYN, Black identified woman)

Clinicians also stressed the need for more upstream policies and interventions to dismantle the historical and pervasive racist structures and practices to improve Black women's care experiences and outcomes.

Although clinicians were aware of structural racism and how it impacts health care practices and behaviors, they struggled identifying how racism impacted their own care practices. A Family Medicine clinician shared:

I think that in our work to undo hundreds and hundreds of years of not just poor healthcare but sometimes purposely damaging healthcare, we've got a lot of work ahead of us, and it's up to us to increase the trust. I don't expect people to come into the healthcare setting trusting us, and so that has to be our work. We have to be proactive, and I'm not exactly sure how to do that. (02-010, Family Medicine, white identified woman)

Overall, clinicians stressed that racism is a multifaceted issue that will require reflexivity among clinicians to recognize and dismantle their racist ideologies, beliefs, language, and behaviors that manifest as acts of anti-racism, health care racism, and obstetric racism that led to inequitable care, in addition to restructuring the current way perinatal care is taught to learners. Additionally, policies and procedures must be implemented to hold clinicians accountable to provide equitable and respectful care to Black women, children, and families.

## Discussion

Three overarching theme emerged from our study supporting the growing literature on obstetric racism. Davis defined obstetric racism as the occurrence and analytic processes, which function at the intersections of medical racism and obstetric violence influencing Black women and birthing people's reproductive care experiences and conditions, resulting in poor health outcomes.^43,46^ Prior research suggests that Black women and birthing people experience maltreatment and preventable adverse outcomes in perinatal health care settings at higher rates than white women.^27^ Findings from our study underscore the mistreatment experienced by Black women in health care settings from the perspectives of perinatal care clinicians and that the root cause is racism.

Participants in our study shared concerns regarding the overreporting of Black women, infants, and family members in relationship to security and police officers in health care settings.^36,47^ There was an overwhelming consensus among these clinicians that Black women and infants receive UTOX screenings at disproportionately higher rates than white women and infants, even when not indicated.^48^ Previous research highlights that Black women are four times as likely to be reported to CPS compared to white women, even though Black women are identified for alcohol/drugs by perinatal care providers at similar rates to white women and are more likely to enter treatment during pregnancy.^49^ Similar to previous research, clinicians from our study also shared that punitive actions extend beyond Black women and infants to their support persons, particularly during birth.^50^

Clinicians in our study highlight the importance of understanding the historical and contemporary impacts of structural racism has on Black women's accessibility to quality care. They stressed that racism is embedded in the curriculum used to teach future clinicians and in the current health care system. They also shared observation of how racism structures “care” hierarchies, whereby Black women experience differential treatment resulting in near-death experiences and other adverse consequences. Clinicians further emphasized the importance of acknowledging and addressing institutional racism in health care settings. They shared the need to restructure medical and nursing school curricula to ensure the next generation is equipped with the skills to provide equitable care to Black women, and policies to hold themselves and their peers accountable.

This study contributes to the growing body of literature importantly examining racism within health care settings. Uniquely drawing from perinatal care clinicians' perspectives, a goal of this research is developing a tailored racial equity training that responds to the needs of perinatal care clinicians. Given the new legislation in California (AB 241) requiring clinicians to take continuing education courses to address implicit bias and mistreatment and improve the quality of care provided to patients, and additional legislation (SB 464) emphasizing improving pregnancy-related care for Black women, it is important to talk with clinicians to understand how racism operates in health care settings from their perspectives to develop trainings that respond to their needs.

Despite this call to action and new legislation, there are no evidence-based training interventions tailored for perinatal care clinicians that have taken a community-based approach to include stories from both Black women and perinatal care clinicians. Data from this study will be used to create vignettes from real-life events, rather than hypothetical situations, allowing clinicians to critically engage with issues that may arise in their health care settings and practice strategies to address them.

The major strength of this study is the diverse sample of perinatal care clinicians we interviewed, enabling us to do an in-depth analysis of perceptions of racism in health care settings from varying perspectives. Limitations include that all clinicians were recruited from California-based health care facilities in the San Francisco Bay Area and were practicing or training in an urban setting. Therefore, study findings are not generalize to more rural settings or among clinicians practicing in states that do not have legislation requiring racial equity training. Also, general emails were sent out to all clinicians and only those who voluntarily responded to participate were included in the study contributing to selection bias. It is possible that clinicians interviewed have an invested interest in advancing racial equity in health care settings.

## Conclusions

Clinicians identified racism as a key factor disrupting optimal prenatal care and birthing experiences for Black women. These clinicians' experiences support the need for a racial equity training to address not only implicit biases but also the history of institutional racism and multilevel tools that clinicians and health care institutions can use to improve Black women's care experiences and outcomes. Our team is using these data to develop a tailored racial equity training uplifting both Black women's and perinatal care clinician's experiences.

## Abbreviations Used

CNM: certified nurse midwives
CPS: child protective services
OB/GYN: obstetricians/gynecologists
UTOX: urine toxicology
